# Supplementary material for: Integrated Analysis of Differential Expression Profiles of miRNA and mRNA in Gonads of Scatophagus argus Provides New Insights into Sexually Biased Gene Expression
Source: Animals (Basel). 2025 May 27;15(11):1564. doi: 10.3390/ani15111564 (PMC12153687; doi:10.3390/ani15111564)
Supplement: Supplementary file 1 [file animals-15-01564-s001.zip › Supplementary Document S1.pdf]

广东海洋大学水产学院实验动物福利伦理审查同意书

Approval of Animal Use Protocol, IACUC, GDOU

批准编号 Approval No.	**GDOU-IACUC-2019-A03004**

本动物实验方案经过广东海洋大学水产学院实验动物伦理委员会审核,符合动物保护、动物福利和伦理原则,符合国家实验动物福利伦理的相关规定。

The animal use protocol listed below has been reviewed and approved by the Institutional Animal Care and Use Committee (IACUC), Fisheries College, Guangdong Ocean University.

**实验名称** Protocol Title	金钱鱼性腺中 miRNA 和 mRNA 差异表达谱的综合分析为了解性别偏向基因表达提供了新见解<br>Integrated Analysis of Differential Expression Profiles of miRNA and mRNA in Gonads of *Scatophagus argus* Provides New Insights Into the Sexually Biased Gene Expression				
**实验申请人** Applicant	雷娅玲<br>Ya-Ling Lei	**职称/学位 Title/Degree**	硕士研究生<br>Master candidate	**邮箱 Email**	leiyaling2022@163.com
**实验负责人** Principle Investigator (PI)	陈华谱<br>Hua-Pu Chen	**职称/学位 Title/Degree**	教授<br>Professor	**邮箱 Email**	chenhp@gdou.edu.cn
**院系(部门)** Department	水产学院<br>Fisheries college		**申请日期** Application date		2019/03/04
**动物种系** Species or Strains	金钱鱼<br>*Scatophagus argus*,		**动物数量** Quantity		20
**计划执行时间** Period of Protocol	2019 年 03 月 04 日至<br>2024 年 12 月 31 日		**实验动物使用许可证** License No. of Laboratory Animal Facility		水产学院<br>Fisheries college
**审查意见** Results of Review	【 √ 】符合动物福利伦理要求，同意实验 **Agree**<br>【 】调整方案后，可进行实验 **Agree after modification**<br>【 】不同意 **Disagree**				
**批准人** Approver	[illegible]		**日期 Date**		2019/03/04

广东海洋大学水产学院

IACUC, Fisheries college of Guangdong Ocean University
