# Supplementary material for: Integrated Analysis of Differential Expression Profiles of miRNA and mRNA in Gonads of Scatophagus argus Provides New Insights into Sexually Biased Gene Expression
Source: Animals (Basel). 2025 May 27;15(11):1564. doi: 10.3390/ani15111564 (PMC12153687; doi:10.3390/ani15111564)
Supplement: Supplementary file 1 [file animals-15-01564-s001.zip › Supplementary Figures S1 and S2.pdf]

Appendix A. Supplementary material

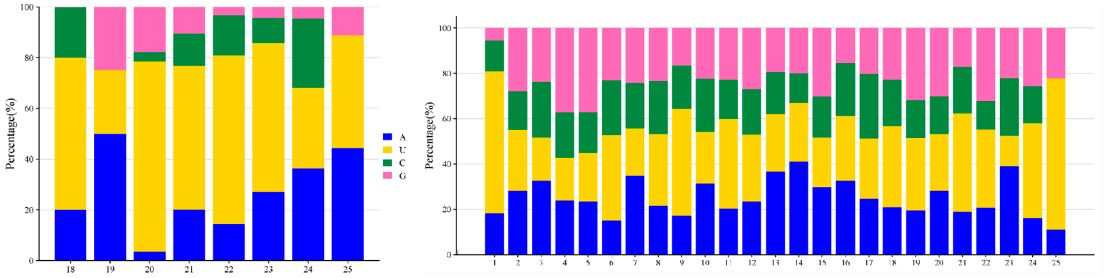

The first base position(left) and each base positionn(right) of know miRNAs

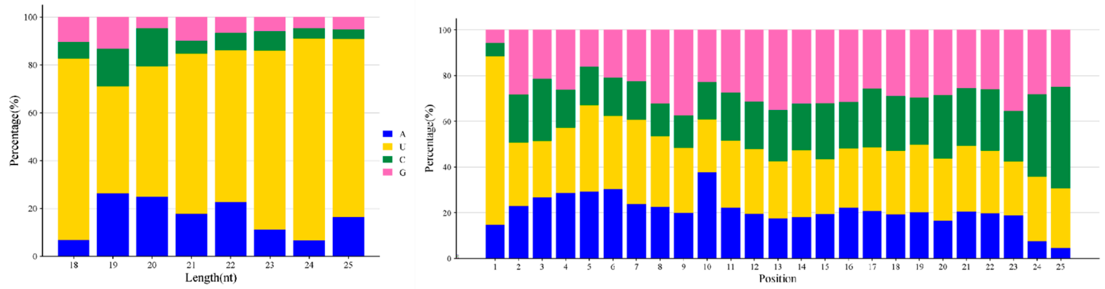

The first base position(left) and each base positionn(right) of novel miRNAs

Figure S1 miRNA nucleotide bias analyses. Base bias percentage on the first position and each position of know miRNAs (top) and novel miRNAs (bottom) specific to *S. argus* sized from 18 to 25 bp, respectively. The x-axis indicates the length of miRNAs and the numbers on the columns are the total number of miRNAs with the specific length of each nucleotide (left). Moreover, the x-axis indicates the location of the base (right). The y-axis indicates the percentage of four nucleotides.

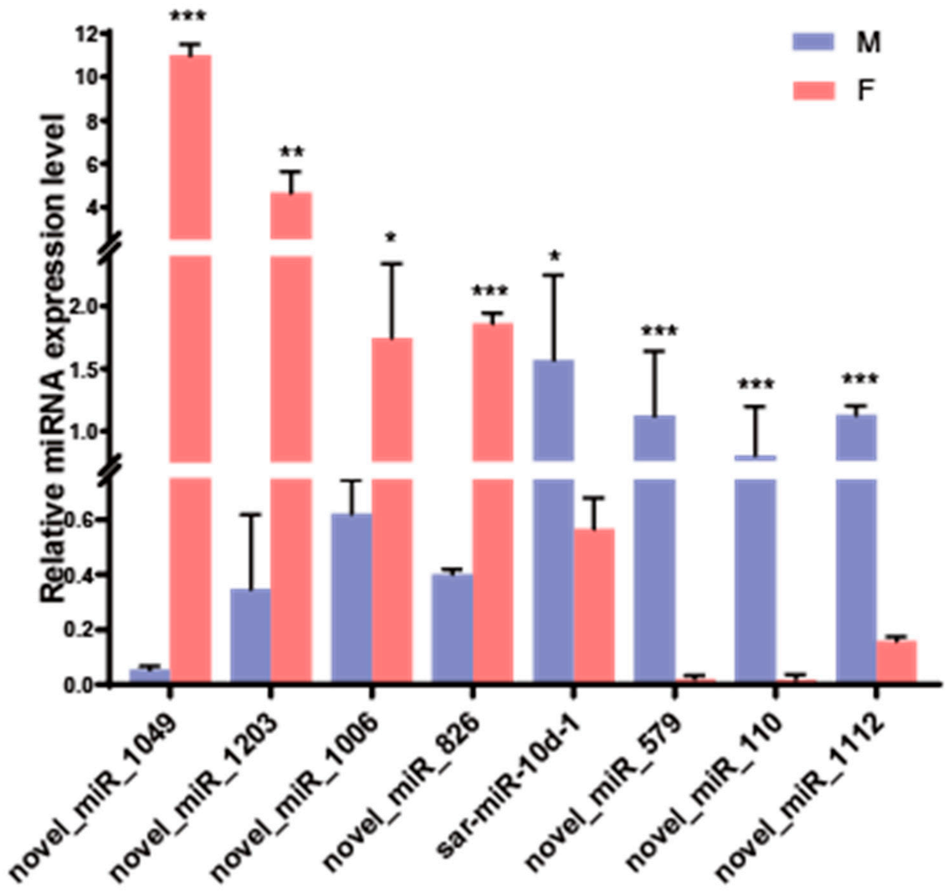

Figure S2 Sexually dimorphic expression of DEMs validated by RT-qPCR. Relative expression levels of eight candidate miRNAs in male (M, blue) and female (F, red) gonads (n = 3 biological replicates). Data are presented as mean ± SD (error bars represent standard deviation of technical triplicates). Statistical significance was determined by two-tailed unpaired Student's t-test with Welch's correction (*$p < 0.05$, **$p < 0.01$, ***$p < 0.001$).
